# Helicity multiplexed broadband metasurface holograms

**DOI:** 10.1038/ncomms9241

**Published:** 2015-09-10

**Authors:** Dandan Wen, Fuyong Yue, Guixin Li, Guoxing Zheng, Kinlong Chan, Shumei Chen, Ming Chen, King Fai Li, Polis Wing Han Wong, Kok Wai Cheah, Edwin Yue Bun Pun, Shuang Zhang, Xianzhong Chen

**Affiliations:** 1 SUPA, Institute of Photonics and Quantum Sciences, School of Engineering and Physical Sciences, Heriot-Watt University, Edinburgh EH14 4AS, UK; 2 School of Physics & Astronomy, University of Birmingham, Birmingham B15 2TT, UK; 3 Department of Physics, Hong Kong Baptist University, Kowloon Tong, Hong Kong; 4 School of Electronic Information, Wuhan University, Wuhan 430072, China; 5 Guangxi Experiment Center of Information Science, Guilin University of Electronic Technology, Guilin 541004, China; 6 Department of Electronic Engineering, City University of Hong Kong, 83 Tat Chee Avenue, Kowloon Tong, Hong Kong; 7 State Key Laboratory of Millimeter Waves, City University of Hong Kong, 83 Tat Chee Avenue, Kowloon Tong, Hong Kong

## Abstract

Metasurfaces are engineered interfaces that contain a thin layer of plasmonic or dielectric nanostructures capable of manipulating light in a desirable manner. Advances in metasurfaces have led to various practical applications ranging from lensing to holography. Metasurface holograms that can be switched by the polarization state of incident light have been demonstrated for achieving polarization multiplexed functionalities. However, practical application of these devices has been limited by their capability for achieving high efficiency and high image quality. Here we experimentally demonstrate a helicity multiplexed metasurface hologram with high efficiency and good image fidelity over a broad range of frequencies. The metasurface hologram features the combination of two sets of hologram patterns operating with opposite incident helicities. Two symmetrically distributed off-axis images are interchangeable by controlling the helicity of the input light. The demonstrated helicity multiplexed metasurface hologram with its high performance opens avenues for future applications with functionality switchable optical devices.

## Abstract (web summary)

The demonstration of visible frequency metasurfaces with broadband optical response is challenging due to plasmonic losses and non-uniform nanofabrication. Here, Wen *et al*. demonstrate a helicity multiplexed metasurface hologram capable of achieving high efficiency and image quality in the visible and near infrared.

## Graphical abstract

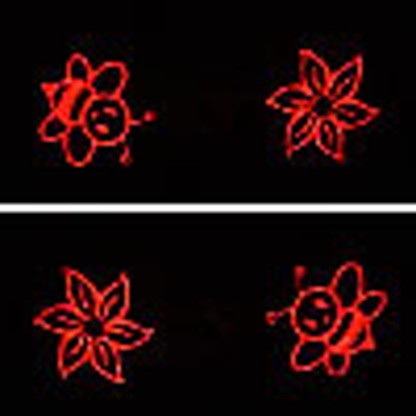

Polarization-selective computer-generated holograms (CGHs) are sensitive to the incident polarization and can separate the readout light by its polarization to reconstruct different holographic images, leading to various applications such as image processing^1^ and multilevel optical switching^2^. The polarization-selective CGHs are traditionally made by etching a birefringent substrate with a surface-relief profile^3^ or an isotropic substrate with sub-wavelength structures to generate effective birefringence^4^. Although birefringent crystals can be used to generate polarization-dependent optical devices^1^,^3^,^5^, the magnitude and form of this birefringence is limited to naturally occurring crystals. Materials with sub-wavelength structures can provide an alternative way towards enhanced and controllable birefringence. However, the extreme anisotropy requires high aspect ratio features, leading to the complexity involved in fabrication since the risks of etching and resolution increase greatly. In addition, the variation in height levels causes shadowing effects that limit the projection angle of the hologram^6^. Furthermore, most of current polarization-selective CGHs suffer from the narrow operating bandwidth as the phase function and the diffraction efficiency change significantly when the incident wavelength deviates from the designed value.

Metasurfaces, the two-dimensional metamaterials^7^,^8^,^9^,^10^,^11^,^12^, have attracted considerable attention of the physics and device research communities owing to a number of unique properties^13^,^14^,^15^,^16^,^17^,^18^,^19^,^20^,^21^,^22^. Metasurfaces have been employed for the application of holography^23^,^24^,^25^,^26^,^27^,^28^,^29^,^30^,^31^,^32^. However, the efficiency of most plasmonic metasurfaces demonstrated so far are extremely low^7^,^14^. To increase the efficiency and bandwidth, a three-layer design consisting of a top layer of metallic antennas, a dielectric interlayer and a continuous metal film at the bottom, has been recently theoretically proposed and experimentally demonstrated in terahertz^33^, infrared and visible ranges^27^,^30^,^31^,^34^,^35^,^36^,^37^,^38^. More recently, Tsai group experimentally demonstrated a four-phase-level CGH with polarization-selective functionality based on this configuration, in which the phase modulation in each pixel is realized by the gold cross nanorods with different sizes^27^. However, each pixel size (1.5 μm) in the experiment is larger than the wavelength, resulting in undesired effects of twin images and multiple diffraction orders. Furthermore, it is practically impossible to generate a continuous phase profile based on this approach as the same size variation causes the unintended amplitude modulation, which deteriorates the image quality. To date, all the demonstrated polarization multiplexed metasurface holograms^27^,^39^ suffer from either low efficiency or poor image quality. Much progress is urgently needed to further expand capabilities and create practical deployable solutions. There are numerous technological or fundamental challenges. Although multi-level phase CGHs can improve the image fidelity, they require delicately controlled geometry to achieve a desired phase profile while maintaining uniform amplitude. On the other hand, more technical difficulties and challenges will be encountered with much stricter requirement of nanofabrication. The experimental demonstration of visible frequency metasurfaces with broadband optical response has been a significant challenge due to the high plasmonic loss and difficulties in high-uniformity nanofabrication.

In this paper, we propose and experimentally demonstrate a helicity multiplexed metasurface hologram (HMMH) capable of achieving high efficiency and high image quality in the visible and near infrared range. To circumvent the problems of low efficiency and poor image quality while maintaining the broadband property, we leverage the recent advances in the realization of high efficiency, broadband reflective-type configuration^31^ and Pancharatnam–Berry phase metasurface to develop easy-to-fabricate and highly efficient HMMHs. In contrast to other types of metasurfaces, a metasurface consisting of nanorods with spatially varying orientation shows superior phase control for the circular polarization and can ease the fabrication. In addition, such a metasurface exhibit a broadband nature since the phase shift is solely dependent on the orientation of the nanorod. Above all, the sign of the acquired phase profile is flipped when the helicity of the incident light passing through the metasurface is changed, offering a new degree of freedom to realize the helicity multiplexed functionality. Unlike the previously demonstrated polarization multiplexed metasurface hologram that is controlled by linear polarization, our holographic images are reconstructed with an incident light with circular polarization. Two symmetrically distributed off-axis images are interchangeable in one identical hologram by controlling the helicity of the input light, that is, right circular polarization (RCP) or left circular polarization (LCP). This work addresses several major issues typically associated with the current polarization multiplexed metamaterial hologram, by virtue of its simplicity, high image quality, high efficiency and broadband nature.

## Results

### Design of the helicity multiplexed metasurface hologram

**Figure 1:**
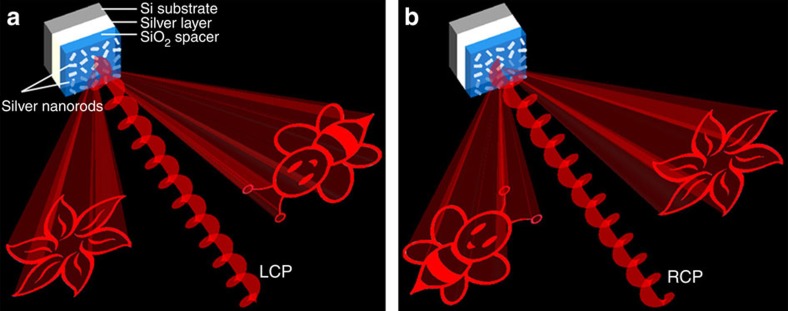
Schematics of the helicity multiplexed metasurface hologram. The reflective-type metasurface consists of silver nanorods with spatially varying orientations on the top, a SiO_2_ spacer (80 nm) and a silver background layer (150 nm) resting on a silicon substrate. (**a**) Under the illumination of LCP light, the holographic images ‘flower' and ‘bee' are reconstructed on the left and right side viewing from direction of the incident light, respectively. (**b**) The positions of the two holographic images are swapped when the helicity of incident light changes from LCP to RCP. The circularly polarized light impinges the reflective-type metasurface at normal incidence and the reflected light that contributes to the images has the same polarization as that of the incident light.

Gerchberg−Saxton algorithm^40^ has long been used for retrieving the phase profile of the phase-only hologram via a propagating function, such as the Fourier transform, if their intensities at their respective optical planes are known. Assuming that the incident light is a uniform planar wave, the phase-only hologram *ϕ*(*x*_0_, *y*_0_) for the generation of the image *I*(*x, y*) in the far field can be obtained, where (*x*_0_*, y*_0_) and (*x*, *y*) represent the hologram plane and the image plane, respectively. Interestingly, we notice that the intensity distribution of the target image *I*(*x*, *y*) becomes *I*(−*x*, −*y*) once the sign of the phase function *ϕ*(*x*_0_, *y*_0_) is flipped to −*ϕ*(*x*_0_, *y*_0_) (Supplementary Note 1), which means that the positions of the two identical images are centrosymmetric (Fig. 1). Hence, it is not surprising to find that the target image at a given region appears or disappears when the sign of the phase profile is flipped, providing an alternative way to realize the image-switchable functionality. If the sign of the phase profile is dependent on the polarization state of the incident light, a polarization multiplexed optical device can be realized.

To realize the polarization-dependent phase profile while maintaining constant amplitude, the reflective-type metasurface adopted here consists of three layers: a two-dimensional array of elongated silver nanorods on the top, a SiO_2_ spacer and a silver background layer on the silicon substrate (shown in Fig. 1a). Although the plasma frequencies of gold and silver lie in the ultraviolet region of the spectrum, the interband transition exerts a huge influence on their properties in the visible range. Gold absorbs most of the blue and green light, while the absorption for silver occurs in the near ultraviolet, which makes silver a very good candidate for visible light. To investigate the performance of the designed metasurface hologram in the visible range, silver is used in our work. After carefully designing the size of the nanorods and the thickness of the SiO_2_ spacer, each nanorod along with the spacer and the background layer function as a reflective-type half-wave plate, whose fast axis is parallel to the long axis of the nanorod (Supplementary Note 2). For the circularly polarized light at normal incidence, the reflected light by a metallic mirror is defined to have the opposite helicity compared to that of the incident light. The reflected light from the nano-waveplate consists of two circular polarization states: one has the same helicity as the incident circularly polarized light but with an additional phase delay and the other has the opposite helicity without the additional phase delay. The additional phase delay is known as Pancharatnam–Berry phase with a value of ±2*ϕ*, where *ϕ* is the orientation angle of each nanorod. Specifically, ‘+' and ‘−' represent the sign of the phase shift for the incident RCP light and that for the incident LCP light, respectively. Hence the reversion of the phase profile can be achieved by either changing the helicity of the circularly polarized light or flipping the sign of the orientation angle *ϕ* of the nanorod for the same circular polarization, offering a new design methodology to achieve helicity multiplexed functionalities.

**Figure 2:**
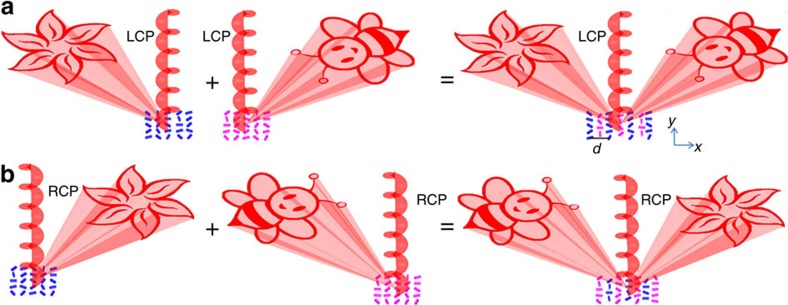
Generation of the helicity multiplexed metasurface hologram. Two sets of hologram patterns are designed to operate with opposite incident helicities and merged together with a displacement vector of (*d*/2, *d*/2). *d* is the distance between neighbouring antennas with a value of 424 nm along *x* and *y* directions. (**a**) Upon the illumination of LCP light at normal incidence, the reconstructed ‘flower' and the ‘bee' are on the left side and on the right side of the incident beam, respectively. The two off-axis images are symmetrically distributed. The blue nanorods and purple nanorods represent the metasurface holograms for ‘flower' and ‘bee', respectively. (**b**) The position for the ‘flower' and that for the ‘bee' are swapped on the illumination of RCP light.

The combination of two sets of hologram patterns operating with opposite incident helicities on the same metasurface is the key step for the realization of the helicity multiplexed functionality. First, Gerchberg−Saxton algorithm is used to generate two phase profiles, which can reconstruct two off-axis images on the different sides of the incident light. Both images (‘bee' and ‘flower') have a projection angle of 22° × 22° and an off-axis angle of 10.35° in the imaging area. Then, the two phase profiles are encoded onto the metasurfaces (Fig. 2a), where the *n*th phase pixel *ϕ*_*n*_ of the hologram is represented by a nanorod with the orientation angle *ϕ*_*n*_/2 defined in the metasurface. After that, two sets of data are merged together with a displacement vector of (*d*/2, *d*/2), as shown in (Fig. 2a). *d* is the distance between neighbouring antennas with a value of 424 nm. Therefore, although the new metasurface contains two sets of hologram data, the size of the sample is still the same and the equivalent pixel size is 300 × 300 nm, leading to the increase of the nanoantenna density. On the illumination of LCP light, the merged metasurface can reconstruct the ‘flower' on the left and the ‘bee' on the right side of the metasurface viewing from the incident beam, respectively (Fig. 2a). Since the sign of the phase profile can be flipped by controlling the helicity of the incident light, the positions of reconstructed images in Fig. 2b are swapped in contrast to those in Fig. 2a when the helicity of the incident light is changed from LCP to RCP. As a result, the reconstructed images on the same position are switchable, that is, either ‘bee' or ‘flower', depending on the helicity of the incident light.

**Figure 3:**
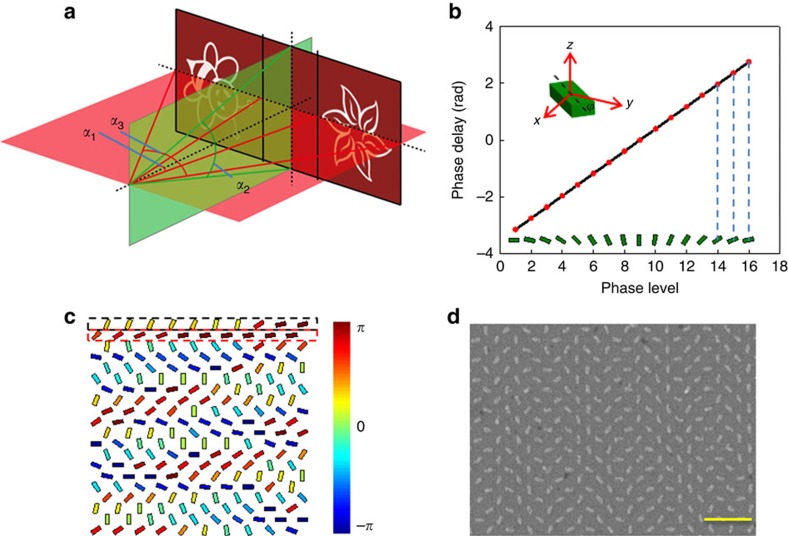
The designed metasurface hologram and the fabricated sample. (**a**) Geometric parameters of the projected images that correspond to the merged hologram. The full off-axis angle *α*_1_, target image angles *α*_2_ and *α*_3_ are designed to be 20.7°, 22° and 64.7°, respectively. (**b**) Phase delay for the different phase levels. Sixteen phase levels (−*π* to *π* with the interval of *π*/8) are used in the design. On each red point, the orientation of the corresponding nanorod is given. The orientation of each nanorod is defined as the angle between the long axis of the nanorod and the *y* axis. (**c**) Schematic of the nanorod distribution for the merged metasurface hologram. The phase levels are denoted by the different colours of the nanorods. The nanorods in the rows with odd number and even number contribute to the reconstruction of ‘bee' and ‘flower', respectively. (**d**) Scanning electron microscopy image of part of the fabricated metasurface. Each nanorod represents a phase pixel defined in the hologram. Scale bar, 1 μm. The equivalent pixel size is 300 × 300 nm^2^.

To reduce the size of the diffraction spots at the image plane, the phase profile of the merged hologram is arranged into a 2 × 2 periodic array with a size of 450 × 450 μm. Each nanorod is 75 nm wide, 200 nm long and 30 nm high. The phase profile is pre-compensated to avoid image distortion because the final projected image has a large field of view of 64.7° × 22° (Fig. 3a). Details about the pre-compensation of the hologram are given in the Supplementary Note 3. Theoretically, the arbitrary phase levels can be easily achieved since the encoding process from the phase profile into pixelated nanoantennas is very straightforward. However, the coupling will be strongest if the corners of the two nearest rods are closest to each other and weakest when they are most apart from each other. To minimize the near field coupling between neighbouring nanorods, we choose 16-phase levels instead of continuous phase distribution (Fig. 3b,c). The phase deviation from the coupling effect of neighbouring nanorods is analysed in the Supplementary Note 4. We use a design that the two reconstructed images appear off-axis to avoid the overlapping between the holographic images and the zero-order spot, dramatically increasing the signal-to-noise ratio (SNR). Standard electron beam lithography and lift-off process are used to fabricate the designed sample. Nanofabrication details are available in the Supplementary Note 5.

### Characterization of helicity multiplexed metasurface hologram

A scanning electron microscopy image of the resulting patterns for HMMH designed at 633 nm is shown in Fig. 3d. A nanorod with an area of 300 × 300 nm^2^ represents a phase pixel defined in the hologram. The required phase profile with 16-phase levels (from −*π* to *π* with an interval of *π*/8) is expected after the incident LCP light beam passes through the fabricated metasurface. The sign of the phase profile will be completely flipped when the helicity of the incident light is changed to the opposite one, leading to the helicity multiplexed functionalities.

**Figure 4:**
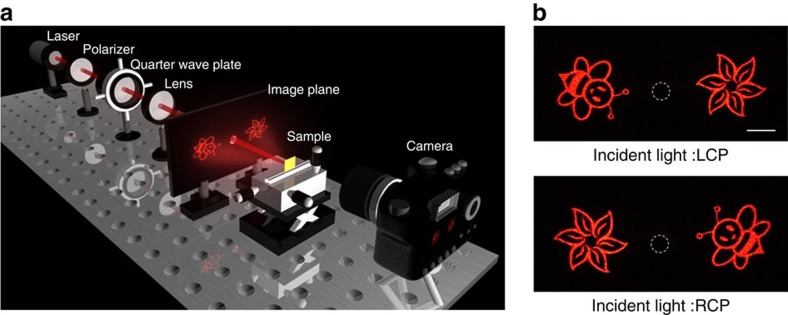
Illustration of the experiment set-up and the experimental results. (**a**) The incident light with various polarization states is obtained by controlling the angle *θ* between the polarization axis of the polarizer and the fast axis of the quarter-wave plate. The incident light impinges normally onto the metasurface and the reconstructed images are projected onto the image plane. The screen is a white paper board with an opening (diameter 6 mm) in the middle, which allows the incident light and zero order reflected light passing through. (**b**) The experimentally obtained images for the incident light with LCP (top) and RCP (bottom). The wavelength of the incident light is 633 nm. The dashed circles mark the edge of the opening. Scale bar, 1 cm.

Figure 4a shows the experimental set-up to characterize the performance of the fabricated metasurface. A laser beam with circular polarization from a tunable laser source (Fianium-SC400-PP) is generated by a polarizer and a quarter-wave plate in front of the sample (Fig. 4a). Then the light is incident onto the fabricated sample, which is mounted on a three-dimensional translation stage, allowing adjustment of the sample position. The incident beam has a radius of ∼1 mm to ensure that the entire metasurface is illuminated by a plane-wave-like wavefront. The reflected holographic image is either projected to a screen in the far field for inspection of the image quality, or focused by a pair of condenser lenses with high numerical aperture to measure the conversion efficiency. The screen is a white paper board with an opening (diameter 6 mm) in the middle, which allows the incident light and zero-order reflected light to pass through. A normal camera instead of a charge-coupled device camera is used to capture images on the image screen.

Figure 4b shows the two reconstructed images on the image plane with a distance of 50 mm between the screen and the metasurface at the wavelength of 633 nm. It should be noticed that the size of the reconstructed images without distortion increase linearly with the reconstruction distance. Two clear images (‘bee' on the left and ‘flower' on the right) with very high fidelity and no distortion (Fig. 4b top) are observed when the metasurface is illuminated with the LCP light. When the light polarization is switched from LCP to RCP, the positions of two images (‘bee' and ‘flower') are swapped (Fig. 4b bottom). In addition, the positions of the two identical images are centrosymmetric, agreeing very well with the theoretical prediction.

**Figure 5:**
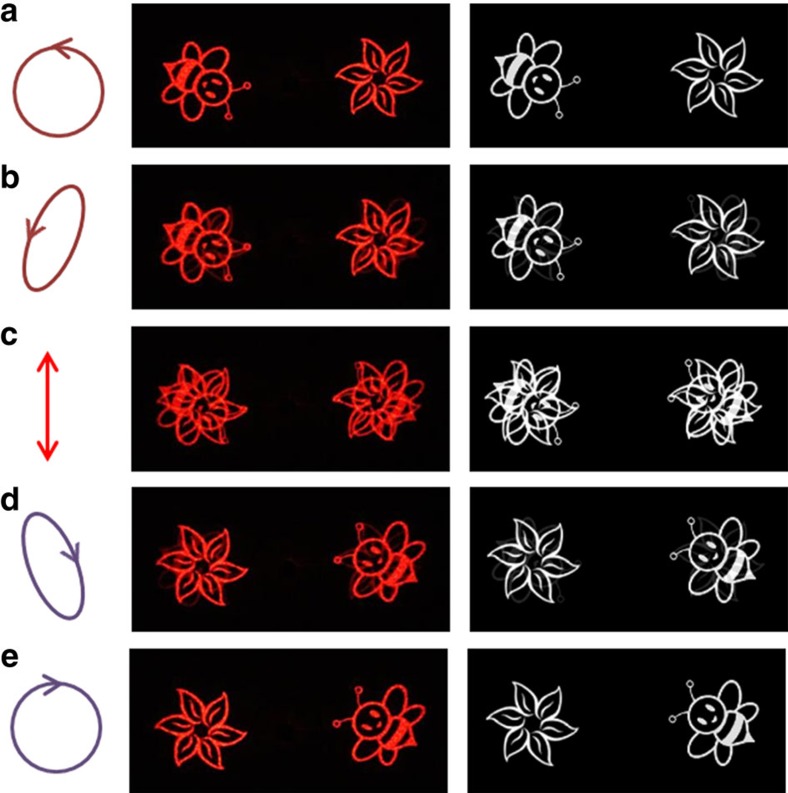
Reconstructed images versus incident polarization states at 633 nm. The polarization states of the incident light are chosen to be (**a**) LCP, (**b**) left-handed elliptically polarized, (**c**) linearly polarized, (**d**) right-handed elliptically polarized and (**e**) RCP. The figures in the middle column and right column represent the experimental results and the corresponding simulation results, respectively.

Two separate images (Fig. 4b) are obtained when the metasurface is illuminated with an input light beam with pure circular polarization (RCP or LCP). However, the two images will overlap upon the illumination of a light beam with elliptical or linear polarization since a completely polarized light beam can be decomposed into a LCP light beam and a RCP light beam. Figure 5 shows the measurement results and simulation results on the reflection side of the metasurface on the illumination of incident light with different polarizations. The intensities of the two overlapping images rise and fall according to the ellipticity of various incident polarized light, which is realized by changing the angle *θ* between the polarization axis of the polarizer and the fast axis of the quarter-wave plate. Initially, the ‘bee' and ‘flower' are reconstructed on the left and right sides of the image plane (Fig. 5a) on the illumination of light with pure LCP. Gradually, we can find two overlapping images with high intensity (‘bee') and low intensity (‘flower') on the left side (Fig. 5b) when the sample is illuminated by a left-handed elliptically polarized light. The intensities of two overlapping images are the same (Fig. 5c) on the illumination of the linearly polarized light since it contains a LCP light beam and a RCP light beam with equal intensities. The intensity of the ‘flower' dominates (Fig. 5d) on the illumination of a right-handed elliptically polarized light. Finally, two separate images are obtained again but their positions are swapped (Fig. 5e) in comparison with those in Fig. 5a. Excellent agreement is seen between the experimental measurements and the simulation results. The quantitative analysis of the energy transfer between the two reconstructed images upon the illumination of incident light with different polarizations is available in the Supplementary Note 6. The evolution process (from ‘bee/flower' to ‘flower/bee') of the reconstructed images can be seen clearly by gradually changing the incident polarization states (Supplementary Movie 1).

## Discussion

Image quality, efficiency and bandwidth are typical issues related to polarization multiplexed CGHs. Although the projected image has a field of view of 64.7° × 22°, the images with very high fidelity and no distortion are observed (Fig. 5a), which benefits from the multiple-level phase and the sub-wavelength pixel size. The SNR is defined as the ratio of the peak intensity in the reconstructed image to the standard deviation of the background noise^25^. The background noise is taken from an area of 100 × 100 pixels excluding the area of the reconstructed image. The SNR for the left side image of Fig. 5a at 633 nm is 43.5. The conversion efficiency is defined as the ratio of the power of the reflected light which forms the holographic images and the input power. Benefiting from the off-axis design, the light that contributes to the ‘bee' and ‘flower' can be collected by using a pair of condenser lenses (*f*=32 mm) and measured separately (*I*_bee_ and *I*_flower_). The conversion efficiency is calculated by *η*=(*I*_bee_+*I*_flower_)/(*pI*_in_), where *I*_in_ and *p* represent the power of the incident light just before impinging on the metasurface and the transmission efficiency of the condenser lenses, respectively. The approach is demonstrated over an ultrabroad spectral range of 475–1,100 nm and the maximum conversion efficiency is 40% at the wavelength of 960 nm.

**Figure 6:**
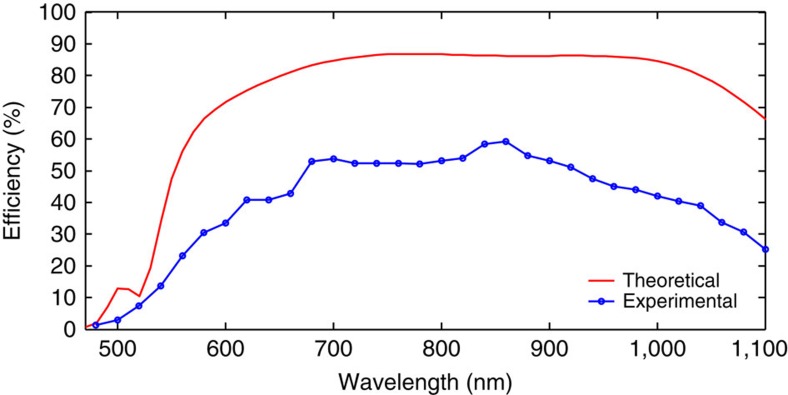
Conversion efficiency of the metasurface hologram. The conversion efficiency is defined as the power of ‘bee' and ‘flower' divided by the power of the incident light. The blue circles represent the experimentally measured efficiencies over a broad range of wavelengths. The titanium layer is not considered in the simulation.

**Figure 7:**
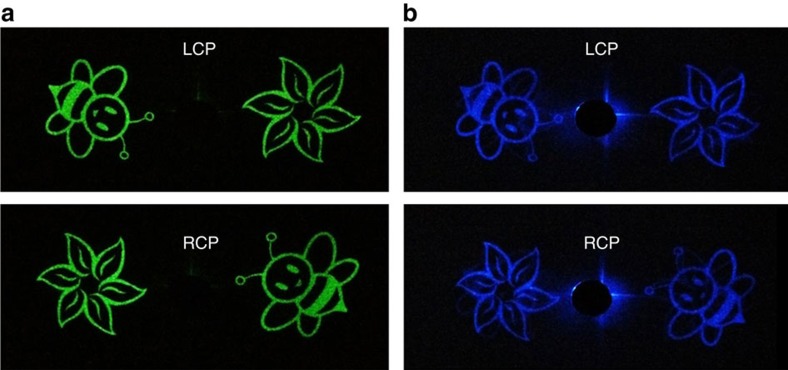
Experimentally obtained images at other visible wavelengths. The wavelengths of the incident light are (**a**) 524 nm and (**b**) 475 nm, respectively.

Although our design is based on off-axis design, the metallic nanorods in a given region not only contribute to its own image but also add noise signal to the other one. Fortunately, the unique nature of Gerchberg−Saxton algorithm and symmetrical distribution of the two patterns enables the optimization of the metasurface design, where each individual nanorod contributes to both images simultaneously. In this design, a target image includes two separated images, each one projected to a different direction. To theoretically explain the performance enhancement of the optimized design, the angular spectrum representation is adopted here to analyze the spatial frequency distribution of the scattered light from these holograms. Details regarding the explanation of the performance enhancement are presented in the Supplementary Note 7. From simulation results, we can clearly see that a significant part of the energy is transferred to high spatial frequencies after the two separate holograms are merged together, while most energy for the optimized design is still allocated to the low spatial frequencies. The propagating wave contributes to the target image while the evanescent wave is absorbed by the metal. Simulation also shows that in our optimized design if we neglect the optical losses, the window efficiency, which is defined as the ratio between the optical power projected into the two image regions and the input power, reaches 90.5%. Based on the optimized design, the experimentally measured SNR in Fig. 5a is increased from 43.5 to 73.8 although our naked eyes can hardly see the difference. Figure 6 shows the experimentally measured conversion efficiency versus wavelength, which is higher than 40% over a broad wavelength band ranging from 620 to 1,020 nm. The maximum conversion efficiency in our experiment is 59.2% at *λ*_0_=860 nm in comparison with the theoretical value of 86% (without titanium layer). The difference between experimental results and simulation results is mainly due to the titanium layer between nanorods and SiO_2_ layer, the fabrication error and measurement method (Supplementary Note 2). The dependence of efficiency and image quality on the incident angle is presented in Supplementary Note 8. The HMMH demonstrated here not only surpasses previously demonstrated polarization multiplexed metasurface holograms^27^,^39^ in terms of performance, but also has advantage over conventional polarization-selective CGHs with only one single lithography step. The realization of metasurface hologram with high image quality in the whole visible wavelength range is rather challenging. However, the metasurface hologram proposed here shows the capability to reconstruct clear images in the visible spectrum. Figure 7 shows the experimentally captured images at the wavelengths of 524 and 475 nm.

From the practical point of view, some distinct properties of the developed HMMHs are of great interest. First, this approach is compatible with standard semiconductor fabrication process. More importantly, the helicity multiplexed functionalities are realized by combining two sets of hologram patterns operating with opposite incident helicities, which not only provides a new methodology to integrate multiple and similar optical functionalities into one single optical element but also can be used to design a single optical element that possesses simultaneously multiple distinct functions. Furthermore, we leverage the recent advances in the realization of high efficiency, broadband reflective-type configuration and Pancharatnam–Berry phase metasurface, which can ensure the high performance of the developed device. The experimentally obtained clear images at the wavelengths of 475, 524 and 633 nm unambiguously show the potential capability of the developed device in the visible range. Last but not least, a new freedom, circular polarization is introduced to realize the polarization multiplexed functionalities, which can have an immediate impact in security and anti-counterfeiting. With the rapid development of hologram technologies, traditional security holograms are not secure any more since they can be easily forged by replicating from a master hologram. The invention of this brand new device will possess a unique advantage over traditional security holograms due to its helicity multiplexed functionalities.

## Conclusion

In conclusion, we have demonstrated a HMMH capable of reconstructing images with high efficiency and high image quality over a wide field of view and a broad range of frequencies. Unlike previously demonstrated polarization multiplexed metasurface holograms that are sensitive to linear polarization, two off-axis images are interchangeable in one identical hologram by controlling the helicity of the incident light. As this approach solves several major issues typically associated with polarization multiplexed CGHs: image quality, efficiency and bandwidth, it paves the way for future practical devices with switchable functionalities that may lead to advances in a wide range of fields such as microscopy, display, security, data storage and information processing.

## Methods

### Design of the metasurface hologram

To generate a target image with a pixel array of *m* × *n* and a projection angle of *α*_*x*_ × *α*_*y*_ in the far field, the period of the hologram *d*_*x*_ and *d*_*y*_ can be calculated by *d*_*x*_*=mλ*/[2tan(*α*_*x*_/2)] and *d*_*y*_*=nλ*/[2tan(*α*_*y*_/2)], respectively. The number of pixels of the hologram is determined by *M=d*_*x*_/Δ*p* and *N=d*_*y*_/Δ*p*, where Δ*p* is the pixel size of the hologram in both *x* and *y* directions. According to the above theoretical models, a phase-only hologram with pixel size of 424 × 424 nm and periods of 225.14 × 225.14 μm is designed by the classical Gerchberg−Saxton algorithm. To reduce the size of the diffraction spots at the image plane, the phase profile of the merged hologram is arranged into a 2 × 2 periodic array with a size of 450 × 450 μm.

## Additional information

**How to cite this article:** Wen, D. *et al*. Helicity multiplexed broadband metasurface holograms. *Nat. Commun.* 6:8241 doi: 10.1038/ncomms9241 (2015).

## Supplementary Material

Supplementary Information Supplementary Figures 1-11, Supplementary Notes 1-8 and Supplementary References.

Supplementary Movie 1 Movie demonstrating the evolution of the reconstructed images.
