# Supplementary material for: Helicity multiplexed broadband metasurface holograms
Source: Nat Commun. 2015 Sep 10;6:8241. doi: 10.1038/ncomms9241 (PMC4579785; doi:10.1038/ncomms9241)
Supplement: Supplementary Information — Supplementary Figures 1-11, Supplementary Notes 1-8 and Supplementary References. [file ncomms9241-s1.pdf]

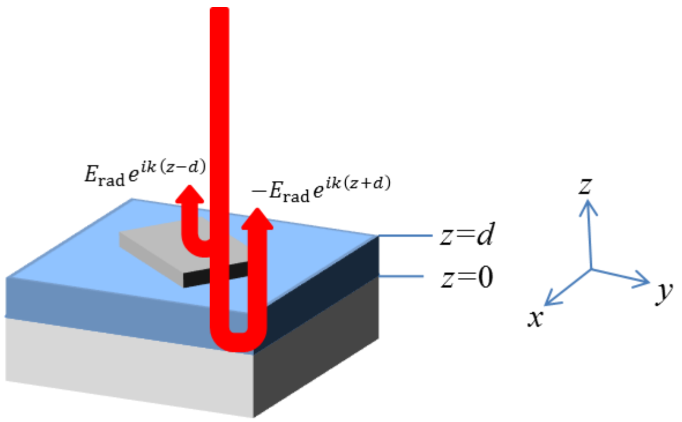

Supplementary Figure 1: Schematic of the nanorod-scattered wave along the +z direction.

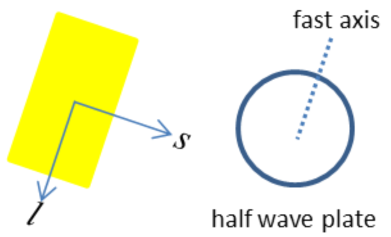

Supplementary Figure 2: The nanorod functions as a half-wave plate. The fast axis of the waveplate is parallel to the long axis of the nanorod.

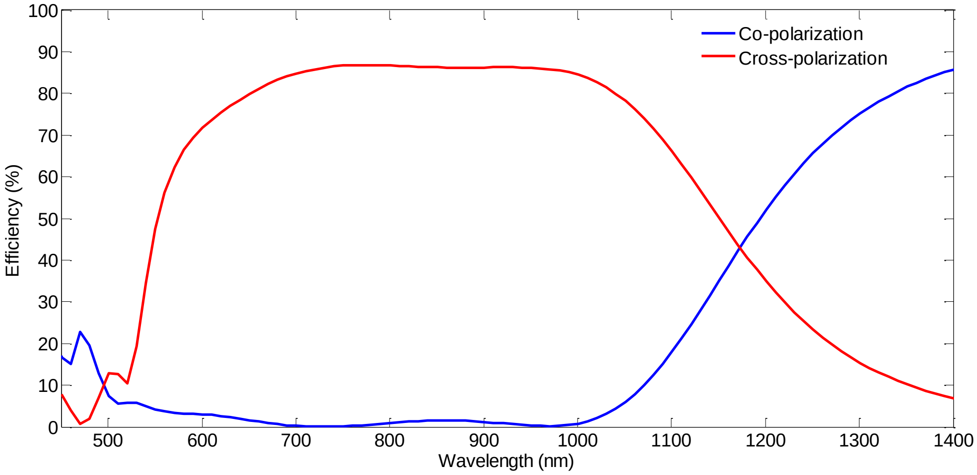

Supplementary Figure 3: The simulated reflectivity curves. The red and blue curves represent the cross-polarized and co-polarized light, respectively. The thickness of the SiO_2 film is 80 nm.

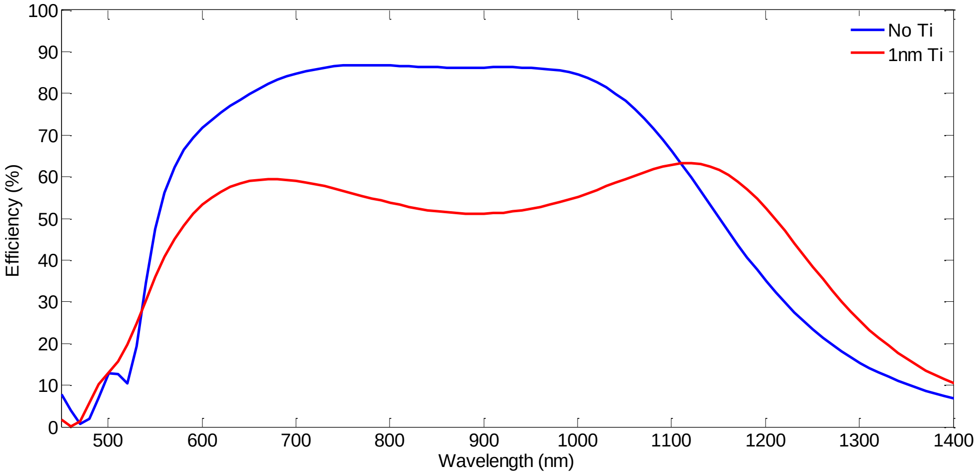

Supplementary Figure 4: The effect of the titanium layer on the reflectivity.

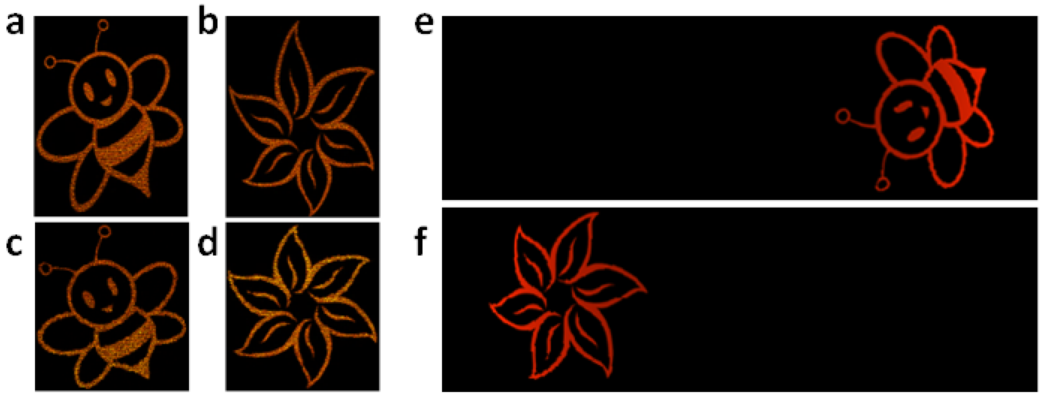

Supplementary Figure 5: Pre-compensation of the hologram. The reconstructed images in (a-b) are without pre-compensation and that in (c-d) are with pre-compensation. (e-f) show the pre-compensated target images for non-paraxial propagation.

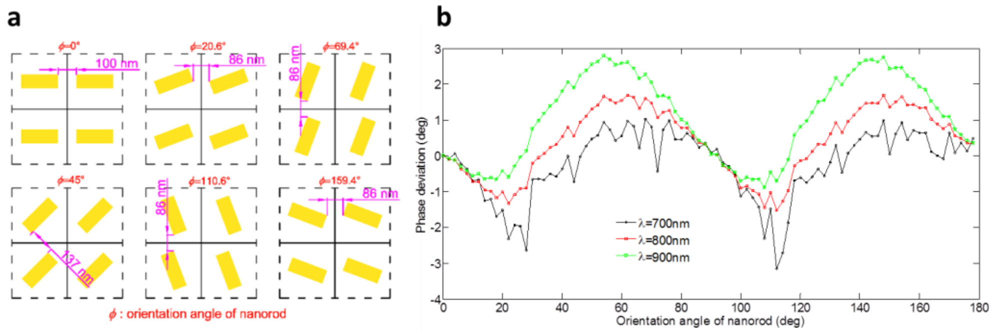

Supplementary Figure 6: Coupling effect of the neighboring nanorods. (a) Schematic demonstration of the uniform array of nanorods with various orientation angles. (b) Simulated phase deviation caused by near field coupling of adjacent nanorods at the wavelengths of 700 nm, 800 nm and 900 nm. The curve shows that absolute value of the deviation is less than 3°.

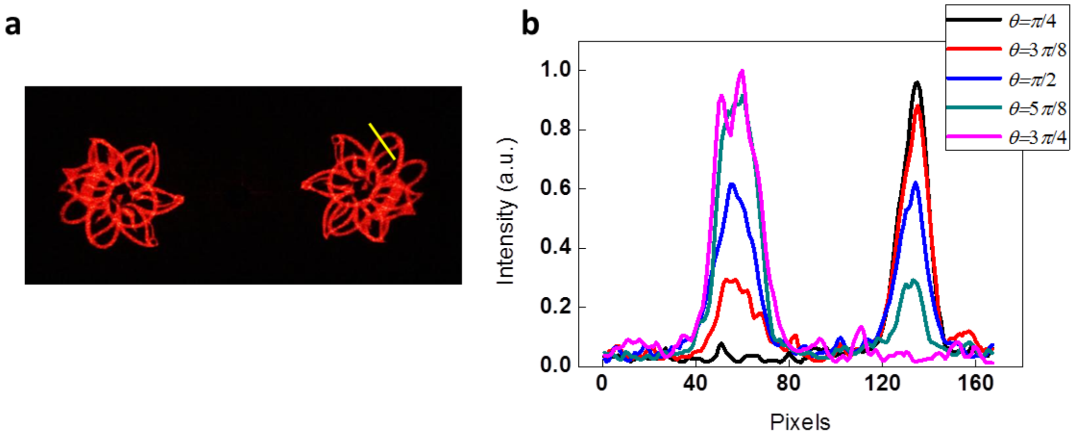

Supplementary Figure 7: Energy transfer between the two reconstructed images. The intensity profiles along a line in the overlapping area of two images are plotted versus polarization states of the incident light at 633 nm. (a) The line that crosses part of the reconstructed 'flower' and 'bee' on the right side of the image plane. (b) The intensity profile along the line in (a) changes with the polarization states of the incident light.

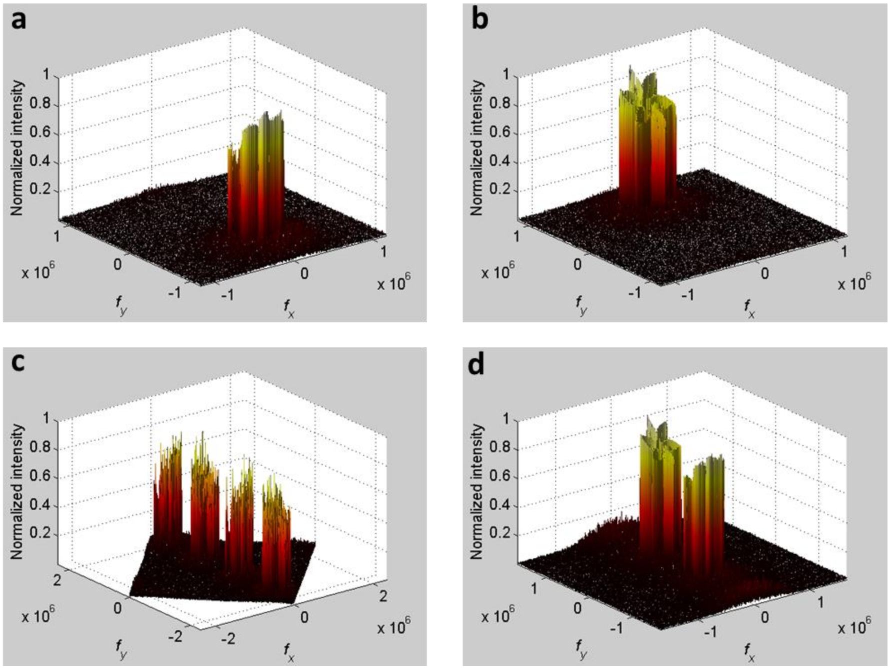

Supplementary Figure 8: Simulated spatial frequency distribution of the scattered light. (a) and (b) show the angular spectrum of the scattered light from the two separated holograms before merging, while (c) and (d) show that for the hologram after merging and the optimized hologram, respectively. The unit of f_x and f_y is m^{-1}.

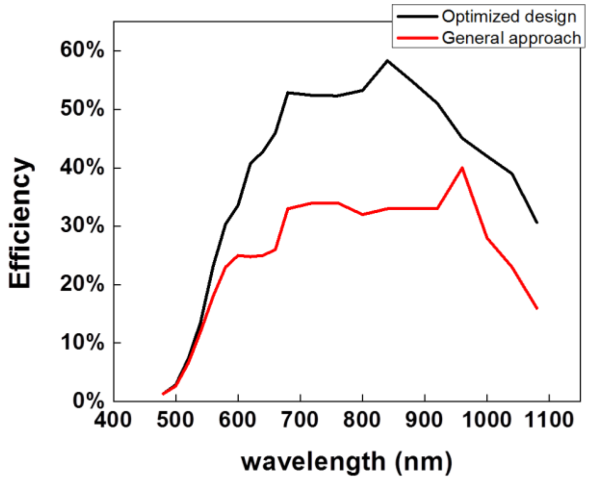

Supplementary Figure 9: Experimentally measured conversion efficiency of the two different designs. The red and black curves show the conversion efficiency of the metasurface at normal incidence based on the general approach and the optimized design, respectively.

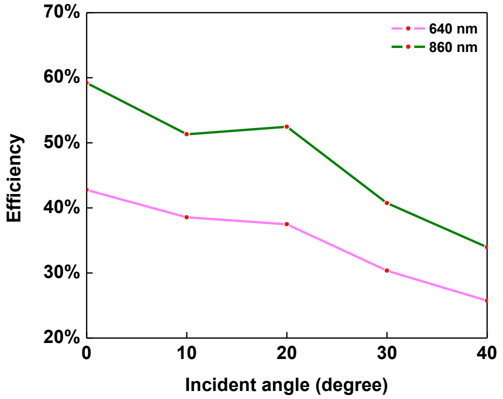

Supplementary Figure 10: The dependence of efficiency on the incident angle.

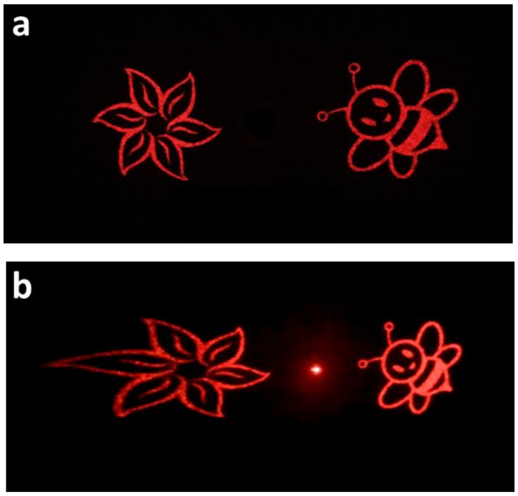

Supplementary Figure 11: The images captured at the incident angle of (a) 0° and (b) 30°.

Supplementary Note 1. Theoretical explanation for the image-switchable functionality

We assume that the incident light is a uniform planar wave with the amplitude A_0, the propagating function is Fourier transformation and the target image has the intensity distribution of $I(x, y)$ in the far field. Based on the Gerchberg−Saxton algorithm[1], the phase-only hologram $\varphi(x_0, y_0)$ can thus been obtained after multiple iterations and we have

$$I(x,y) = A_0^2\left|F[e^{i\varphi(x_0,y_0)}]\right|^2 \tag{1}$$

F represents the Fourier transformation. If the phase distribution of the hologram $\varphi(x_0, y_0)$ is changed into $-\varphi(x_0, y_0)$, the intensity distribution changes into

$$I'(x,y) = A_0^2\left|F\left[e^{-i\varphi(x_0,y_0)}\right]\right|^2 \tag{2}$$

$$= A_0^2\left|\iint_{-\infty}^{\infty} e^{-i\varphi(x_0,y_0)}e^{-i2\pi(f_x x_0+f_y y_0)}\,dx_0dy_0\right|^2 \tag{3}$$

$$= A_0^2\left|\left[\iint_{-\infty}^{\infty} e^{i\varphi(x_0,y_0)}e^{-i2\pi(-f_x x_0-f_y y_0)}\,dx_0dy_0\right]^*\right|^2 \tag{4}$$

$$= I(-x,-y) \tag{5}$$

Where $f_x=x/\lambda z$ and $f_y=y/\lambda z$ (λ is the wavelength and z is the reconstruction distance) in Eqs. (3)-(4). The intensity distribution for target image $I(x, y)$ is changed into $I(-x, -y)$ once the sign of the phase function $\varphi(x_0,y_0)$ is flipped to $-\varphi(x_0,y_0)$, which means that the positions of the two identical images are centrosymmetric, providing an alternative way to realize the image-switchable functionality.

Supplementary Note 2. The reflective-type metasurface

2.1. Broadband characteristic

The broadband characteristic of the reflective type metasurface can be explained by using Jiang's model for reflective-type metallic nanostructure array[2]. Assuming that the light scattered by the nanorod has the amplitude of E_{rad} at $z=d$, then the scattered light propagating along the $+z/-z$ has the expressions of (Supplementary Fig. 1)

$$E_{\mathrm{rad}} e^{ik(z-d)} \tag{6}$$

and

$$E_{\mathrm{rad}} e^{-ik(z-d)} \tag{7}$$

The silver background layer functions like a perfect mirror. After a round trip through the dielectric layer, the formula (7) changes into

$$-E_{\mathrm{rad}} e^{ik(z+d)} \tag{8}$$

The total scattered light propagating along the $+z$ direction is expressed as the superposition of (6) and (8)

$$E_{\mathrm{scatter}} = E_{\mathrm{rad}} e^{ik(z-d)} - E_{\mathrm{rad}} e^{ik(z+d)} = E_{\mathrm{rad}} e^{ikz}\left(e^{-ikd} - e^{ikd}\right) \tag{9}$$

By properly choosing d, the dispersion of E_{rad} can be cancelled out by the dispersion of $\left|e^{-ikd} - e^{ikd}\right|$, hence the E_{scatter} can maintain its amplitude over a wide range of wavelengths.

2.2. High efficiency characteristic

The three-layer structure functions like a Fabry-Pérot–like cavity[3], where the thickness d of the dielectric spacer corresponds to the cavity length. By choosing d (80 nm in our experiment) and the geometry of the nanorods carefully, the superposition of these l polarized reflected fields results in a constructive interference and gives a near-unity overall reflection, so does the s polarized fields (Supplementary Fig. 2). Moreover, the s polarized component is added a phase delay of π versus the l polarized component[4]. As a

result, each nanorod along with the spacer and the background layer functions as a reflective-type half-wave plate. The nanorod cell is designed and simulated by CST microwave studio software. The simulation method we used to calculate the reflectivity curves of co-polarization and cross-polarization is same as that in reference[4]. The refractive indices of SiO_2 and silicon we use are 1.45 and 3.45, respectively. The refractive indices of silver and titanium are taken from the reference[5].

When the incident light is circularly polarized, the reflectivity for the co-polarized and cross-polarized light is calculated as shown in Supplementary Fig. 3. This figure shows that most of the incident light is transformed by the metasurface and preserves the polarization of the incident light within a broad range of wavelengths.

The difference between experimental results and simulation results is mainly due to the titanium layer between nanorods and SiO_2 layer, the fabrication error and measurement method. To improve the adhesion of silver to SiO_2 in our fabrication process, a titanium layer (~1 nm) is added between nanorods and SiO_2 layer, which has a huge impact on the conversion efficiency. The difference with and without titanium layer can be clearly seen in Supplementary Fig. 4. Of course, the titanium layer with a thickness less than 1 nm is not a uniform film, but isolated particles. Therefore the theoretical model used for the simulation of titanium layer is not very accurate. Apart from that, the fabrication error (e.g., missing nanorods and nanorod distortion) also plays an important part in its performance. Furthermore, our design is based on normal incidence. However, in order to measure the conversion efficiency, a lens with a focal length of 30 cm is used to shrink the beam size of the incident light since it is larger than the sample size, meaning that the incident light is not a real plane wave. In addition, the holographic image from the

metasurface hologram is projected into a broad angular range (64.7°×22°) in the experiment. The broad angle scattering is also expected to induce the lower peak reflection than the calculated results.

Supplementary Note 3. Pre-compensation of the hologram

As the designed CGH is expected to create a wide image angle of 64.7°×22°, there is pattern geometrical distortion (see Supplementary Fig. 5(a) and (b)) without pre-compensation of the hologram. In order to obtain an image with uniform intensity and without distortion in the far field ((see Supplementary Fig. 5(c) and (d)), the intensity distribution of the target image is modified for non-paraxial propagation. As shown in Supplementary Fig. 5(e) and (f), the intensity at the edges of the target images has been enhanced to compensate the inhomogeneous scattering of nanorods. Due to the large angular range, the Rayleigh-Sommerfeld diffraction method is used to simulate the holographic image[6].

Supplementary Note 4. Phase deviation from the coupling effect of neighbouring nanorods

For a given antenna in the metasurface, the effect of the near field coupling from its neighbouring nanorods can be evaluated by the phase deviation from its designed value (2φ, φ is the orientation angle). For simplicity, the deviation is simulated for a uniform array of nanorods but with different orientations (Supplementary Fig. 6(a)). The nearest distance between the two neighboring antennas is 86 nm when φ equals to 20.6°, 69.4°, 110.6° and 159.4°, which correspond to the four peaks of the phase deviation curve. Supplementary Fig. 6(b) also shows that the maximum phase deviation is small (±3°

maximum) for several wavelengths, which is much less than the phase step (16 steps, 22.5°/step) used in our hologram design.

Supplementary Note 5. Fabrication of the designed metasurface

To fabricate the designed nanoantenna structures, the standard electron-beam lithography (EBL) and lift-off process are used. Firstly, the silver background layer (150nm) and the SiO_2 spacer (80 nm) are deposited onto the silicon substrate by using the electron beam evaporator. Then, the positive poly methyl methacrylate (PMMA) resist film is spin coated on the SiO_2 spacer layer and baked at 180℃ for two minutes. Then, the nanostructures are defined on the PMMA film by EBL. Prior to silver deposition, a titanium layer is deposited on the silicon dioxide to improve the adhesion of silver to SiO_2. After that, a 30 nm silver film is deposited on the sample via thermal evaporation. Finally, the metasurface structure is achieved by a subsequent lift-off procedure. The shapes of the nanorods are slightly distorted due to the fabrication error in the lift-off process.

Supplementary Note 6. Quantitative analysis of the energy transfer between the two reconstructed images upon the illumination of incident light with different polarizations

To quantitatively analyze the energy transfer between the two reconstructed images upon the illumination of incident light with different polarizations, the intensity profiles along a line in the overlapping area of two images are given in Supplementary Fig. 7, which further confirms the theoretical analysis. Different incident polarization is achieved by changing the angle θ formed between the polarization axis of the polarizer and the fast axis of the quarter wave plate. θ is chosen at five separate values, i.e., $\pi/4$, $3\pi/8$, $\pi/2$, $5\pi/8$

and $3\pi/4$.The horizontal axis and the vertical axis represent the pixel numbers along the line and the corresponding normalized pixel intensities, respectively. The two peak positions around 55th and 135th pixels represent the parts that belong to 'bee' and 'flower', respectively.

Supplementary Note 7. Optimized design of the metasurface hologram

The combination of two sets of hologram patterns operating with opposite incident helicities on the same metasurface is a design methodology we proposed here to realize image-switchable functionality. However, there is crosstalk between two sets of holograms after combining them together. Fortunately, this design can be further optimized since the two phase profiles are generated by using same iterative method. A more straightforward design approach is to provide a target image that includes two separated images, each one projected to a different direction.

To theoretically explain the performance enhancement of the optimized design, the angular spectrum representation is adopted here to analyze the spatial frequency distribution of the scattered light from these holograms. By using angular spectrum representation, a monochromatic field of the wavelength λ can be described as a superposition of a series of plane waves and evanescent waves[7], whose spatial frequencies in the x and y directions are represented by f_x and f_y, respectively. (f_x, f_y) pair represents a propagating wave if $f_x^2+f_y^2 \leq 1/\lambda^2$, or an evanescent wave if $f_x^2+f_y^2>1/\lambda^2$. The propagating wave contributes to the target image while the evanescent wave is absorbed by the metal.

Benefiting from the geometric berry phase[8], the phase profile of the metasurface hologram $\varphi(x, y)$ is dispersionless. As a result, the metasurface hologram can produce a

wavefront with the prescribed phase distribution of $\varphi(x, y)$ regardless of the wavelengths. The fast Fourier transformation is then applied to obtain the normalized angular spectrum of the complex field representing the wavefront. The normalized angular spectrum corresponding to the two separated holograms before merging, after merging and the optimized hologram are shown in Supplementary Fig. 8. By comparing Supplementary Fig. 8(a) and 8(b) with 8(c), it shows that a significant part of the energy is transferred to high spatial frequencies ($|f_y|>10^6$ m^{-1}) after merging the two separate holograms together. However, in the optimized design, most energy is still allocated to the low spatial frequencies ($|f_y|<10^6$ m^{-1}) as shown in Supplementary Fig. 8(d). For the incident wavelength of 475 nm-1100 nm, the calculation results show that a considerable part of waves with high spatial frequencies are evanescent waves for the sample based on the general design approach (Supplementary Fig. 8(c)). The proportion of the evanescent waves increases with the incident wavelength. In contrast, the majority of the energy for the optimized design corresponds to propagating waves and contributes to the target image (Supplementary Fig. 8(d)). As a result, the performance of the metasurface hologram based on the optimized design is improved. Experimental results have confirmed our prediction (see Supplementary Fig. 9). Of course, as each nanorod defined in the metasurface is affected by the coupling from other nanorods, it might not be very accurate to consider each nanorod in the metasurface as an isolated phase pixel in the hologram. However, full wave numerical simulation of our hologram is beyond our simulation capability due to the large array of antennas contained in the hologram.

Supplementary Note 8. The dependence of efficiency and image quality on the incident angle

The metasurface hologram is designed for the incident light at normal incidence. Therefore, the performance of the hologram will change when the light impinges the sample at oblique incidence. The experimentally measured efficiency versus incident angle is shown in Supplementary Fig. 10, which clearly shows that efficiency degrades with the increase of the incident angle. Furthermore, the reconstructed images are distorted to some extent since the phase profile of the scattered light deviates from its designed distribution (see Supplementary Fig. 11).

Supplementary References

1. Gerchberg, R. W., Saxton, W. O. A Practical Algorithm for the determination of phase from image and diffraction plane pictures. *OPTIK* **35**, 237-246 (1971).
2. Jiang, S. C., *et al.* Tuning the polarization state of light via time retardation with a microstructured surface. *Phys. Rev. B* **88**, (2013).
3. Grady, N. K., *et al.* Terahertz metamaterials for linear polarization conversion and anomalous refraction. *Science* **340**, 1304-1307 (2013).
4. Zheng, G., Mühlenbernd, H., Kenney, M., Li, G., Zentgraf, T., Zhang, S. Metasurface holograms reaching 80% efficiency. *Nat. Nano.***10**, 308-312 (2015).
5. Palik, E. D., Handbook of Optical Constants of Solids, *Academic Press*, New York (1998).
6. Shen, F., Wang, A. Fast-Fourier-transform based numerical integration method for the Rayleigh–Sommerfeld diffraction formula. *Appl. Opt.* **45**, 1102–1110 (2006).
7. Novotny, L., Hecht, B. Principles of nano-optics, *Cambridge university press*, New York (2012).
8. Huang, L. et al. Dispersionless phase discontinuities for controlling light propagation. *Nano Lett.* **12**, 5750–5755 (2012).
